# Health Risk Assessment of Trace Metals in Various Environmental Media, Crops and Human Hair from a Mining Affected Area

**DOI:** 10.3390/ijerph14121595

**Published:** 2017-12-18

**Authors:** Wushuang Xie, Chi Peng, Hongtao Wang, Weiping Chen

**Affiliations:** 1 State Key Laboratory of Urban and Regional Ecology, Research Center for Eco-Environmental Sciences, Chinese Academy of Sciences, No. 18, Shuangqing Road, Beijing 100085, China; wsxie_st@rcees.ac.cn; 2 University of Chinese Academy of Sciences, No. 19, Yuquan Road, Beijing 100049, China; wanghongtao90@163.com; 3 Institute of Environmental Engineering, School of Metallurgy and Environment, Central South University, Changsha 410083, China; chipeng@csu.edu.cn

**Keywords:** trace metals, multi-media, hair, risk assessment, exposure assessment

## Abstract

Long term exposure to trace metals in various media is of great concern for people living in known pollution sources, such as mining and industrial activities. Health risk assessment and human hair analysis can provide important information for local environmental management. Information on distribution characteristics of trace metals in soil, water, sediment, air, local crops, and human hair from a typical mining area in southern China was collected. Results show there exists severely trace metal contamination in soil, sediment, and air. Arsenic and Pb contents in the local children’s hair are higher than the upper reference values, and the accumulation of residents’ hair trace metals shows great correlation with the ingestion and inhalation pathways. Arsenic contributes 52.27% and 58.51% to the total non-cancer risk of adults and children, respectively. The cancer risk of Cd in adults and children are 4.66 and 3.22 times higher than the safe level, respectively. Ingestion exposure pathway of trace metals largely contributes to the total non-cancer and cancer effect. The metals As, Cd, and Pb are major risk sources and pollutants that should be given priority for management, and ingestion pathway exposure to trace metals through soil and crops should be controlled.

## 1. Introduction

Like many other developing countries, such as Congo, Chile, and Vietnam, China has been suffering from serious pollution caused by trace metals [1,2,3,4,5]. Rapid development of industrialization and urbanization in these countries has accelerated the release of trace elements from traffic, fuel components, and other non-specific sources [6,7,8]. Additionally, industrial and mining activities are one of the most important trace metal pollution sources in the environment [9,10,11]. After releasing into the environment, trace metals can accumulate in different environmental medias in many chemical forms [12] and damage human health through various absorption pathways, such as the soil-food chain, inhalation, and oral intake [13,14,15]. Even at low concentrations, these contaminations are toxic to human health, especially to children’s health. Therefore, trace metals exposure has been attracted great attentions [16,17,18,19,20,21].

Human hair has been considered as a suitable indicator for estimating long-term metal exposure in China [22,23] and other countries [24,25,26]. The comprehensive analysis of the content of trace elements in human hair, not only reflects the overall characteristics of regional geochemistry, but also can reflect the metabolic status and health level of life elements in different people. Although there are many researches about the trace metals levels of exposed residents’ hair, studies on the influencing factors about the accumulation of metals in hair are relatively lacking.

Serious systemic health problems can develop as a result of excessive dietary accumulation of trace metals in the human body [17]. The hazard quotient (HQ), formalized by the U.S. EPA (Environmental Protection Agency), has been widely utilized to evaluate the potential health risks that are associated with long-term exposure to metals in various media, including soil, drinking water, ambient air, and food [27]. There are many human health risk assessment of trace metals through various exposure pathways [16,28,29], but the reports about different media and systematic multi-pathways analysis are rare. Limited work has been reported on the correlation between trace metals exposure pathway and residents’ hair.

In this study, a farmland closing to the Dabaoshan mining area, Southern China is selected as a case to assess the potential health risks posed by trace metal exposure to multiple media and the accumulation of trace metals in hair of local dwellers. The study site has experienced substantial uncontrolled development over the past 30 years, with the expansion of mining and industrial activities. Large amounts of metals were released through by sewage, atmospheric deposition, or solid wastes, resulting in serious metal pollution. The specific objectives of this study are: (1) to explore the distribution of trace metal in different environmental media under long term mining and industrial activities; (2) to study the concentrations of trace metals in the hair of residents and influencing factors; and, (3) to determine the potential health risk of trace metals via the multiple routes of ingestion, inhalation, dermal exposure, and diet from the soil-crop-water-air system.

## 2. Materials and Methods

### 2.1. Study Area

**Figure 1:**
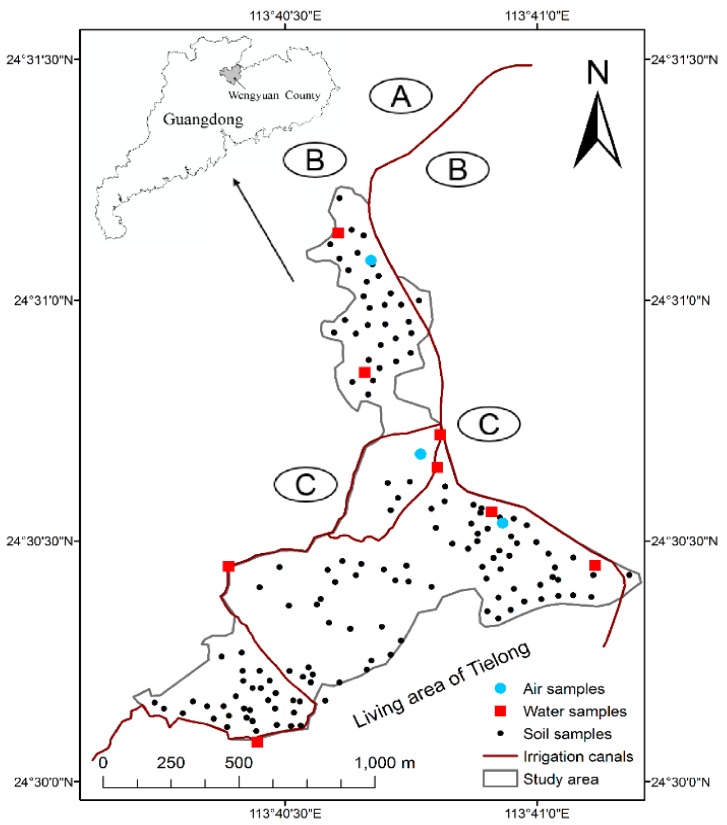
Location of study area and sampling sites.

The study locates in Tielong town, Shaoguan city, Guangdong province (24°5′ N, 113°68′ E), covering a total area of 96.5 km^2^ and having a total population of 5962 inhabitants (Figure 1). There were a non-ferrous metals company (A) and mining company (B) built on the north of the Tielong town, companies has been operating since 1980s, but are now closed, large amounts of tailings were produced during mining in the Zn-Cu poly-metallic deposit. A cement plant (C) is located in the middle of the town. The township has two irrigation canals, one of the canals flows down the mine and was once used for agricultural irrigation.

### 2.2. Samples Collection and Analysis

Various media samples, including 145 surface soil samples (0–20 cm), 24 surface water samples, 6 sediment samples, and 12 air deposits were collected to obtain the mean and ranges of trace metal concentrations in the study area.

Each soil sample was the mixture of nine subsamples around the designated location. The soil samples were air-dried, picked for residues/stones, and sieved twice using a 2- and 0.149-mm mesh. Of the sieved soil sample, 0.25 g was digested in a mixture of HCl-HNO_3_-HClO_4_-HF, until the solution was translucent or reduced to about 2–3 mL. The final solution was diluted to 50 mL, using double-distilled water (ddH_2_O).

Atmospheric depositions were collected from three sampling sites in the study area. A total 12 samples of air samples were collected monthly with membrane filter, by using an air sampler with a larger volume (20 cm × 38.5 cm). Samples were digested with an HCl-HNO_3_-HF-HClO_4_ solution for the metal concentrations.

A total of 24 irrigation water samples were collected in 1 L acid-washed polyethylene bottles from eight sites at random to represent the entire area. Microwave digestion of water samples for metal analysis was done by measuring 50 mL into 5 mL HNO_3_.

Six different types of plant species were harvested from the study area. The edible portions of the plants were harvested for trace metal analysis. The six species plants were cabbage (*n* = 12), oil wheat (*n* = 6), tomato (*n* = 10), radish (*n* = 15), corn (*n* = 14), and pepper (*n* = 3). Plant samples were washed by using ddH_2_O and were dried in a hot air oven at 75 °C for 24 h. The dried residues were mashed into fine particles before microwave digestion, 0.2 g of mashed samples were digested with 8 mL of HNO_3_ and H_2_O_2_ with 3:1 ratio.

Hair samples were collected from 76 farmers in Tielong town who settled in the town, with a residence time of at least 10 years (except children). Farmers with a history of trace metal exposure from other sources rather than the study area were not included in the study, the hair was selected after the natural growth of the occipital without heat dyed. Hair samples from human subjects were collected after informing farmers of the purpose of the research and subsequently soliciting consent from farmers. Hair samples were washed with neutral detergent and three times with ddH_2_O and put in a drying furnace at 70 °C over night. After cooling, the samples were caught in small pieces of 2 mm length and 0.1 g of hair samples was weighted, mixed with 6 mL HNO_3_ and 1 mL HClO_4_.

The concentrations of As, Cd, Cr, Cu, Ni, Pb, Zn in the samples were analyzed by inductive coupled plasma mass spectrometer (ICP-MS) (7500a, Agilent Technologies Inc., Santa Clara, CA, USA). All of the experiments were conducted within strict experiment conditions to eliminate/minimize possible contamination and interference. Standard reference samples (including GSS-5, GSB-26, and GSH-1) and blank samples were used for quality control and recovery analysis. Recovery rates for metal contents were controlled between 80% and 120%.

### 2.3. Daily Intake (DI) of Trace Metals

The daily intake (DI) of trace metals depended on both the trace metal concentration in crops and the amount of consumption of crops [30]. The DI for human were determined by:

(1) $$\mathrm{DI}=C_{m}\times C_{f}\times W_{f}$$

where $C_{m}$ (mg·kg^−1^, on dry weigh basis) is the concentration of trace metals in crops. $C_{f}$ represents conversion factor, 0.085 is used to convert dry weight to fresh weight. $W_{f}$ (kg·(person·day)^−1^) represents the average daily consumption of crops, the average value for adults and children are considered 0.345 and 0.232 kg·(person·day)^−1^ [30,31]. The trace metal intakes are compared with the provisional tolerable daily intake (PTDI) [32,33].

### 2.4. Risk Assessment

Currently, there is no agreed limit for acceptable maximum carcinogenic and non-carcinogenic risk levels in China, we therefore employed the U.S. EPA model and their threshold values in order to assess the potential human health risk that is posed by trace metal pollution in the study [34]. Six exposure routes are considered, including: (1) direct ingestion of soil particles; (2) dermal contact with soil particles; (3) diet through the food chain; (4) inhalation of soil particles from the air; (5) oral intake from groundwater; and (6) dermal intake from groundwater [17].

The calculation for the average daily dose of contaminants via various exposure pathways are as follows [35,36]:

(2) $${\mathrm{ADD}}_{\mathrm{ing}}=\frac{C\times \mathrm{IngR}\times \mathrm{EF}\times \mathrm{ED}\times \mathrm{CF}}{\mathrm{BW}\times \mathrm{AT}}$$

(3) $${\mathrm{ADD}}_{\mathrm{inh}}=\frac{C\times \mathrm{InhR}\times \mathrm{EF}\times \mathrm{ED}}{\mathrm{PEF}\times \mathrm{BW}\times \mathrm{AT}}$$

(4) $${\mathrm{ADD}}_{\mathrm{derm}}=\frac{C\times \mathrm{SA}\times \mathrm{AF}\times \mathrm{ABS}\times \mathrm{EF}\times \mathrm{ED}\times \mathrm{CF}}{\mathrm{BW}\times \mathrm{AT}}$$

where C is the metal concentration in the samples, IngR is the ingestion rate, InhR is the inhalation rate, PEF is the particle emission factor, SA is the surface area of skin exposed to pollutants, AF is the skin adherence factor, ABS is the dermal absorption factor, EF is the exposure frequency, ED is the exposure duration, BW is the body weight, and AT is the average time for non-carcinogens or carcinogens, CF is the units conversion factor.

Both the carcinogenic and non-carcinogenic risk for all of the metals through ingestion, inhalation, dermal, and diet exposure pathways were calculated. The non-carcinogenic risk from individual trace metals can be expressed as the hazard quotient (HQ).

(5) $${\mathrm{HQ}}_{i}=\overset{3}{\underset{j=1}{\sum }}\frac{{\mathrm{ADD}}_{\mathrm{ij}}}{{\mathrm{RfD}}_{\mathrm{ij}}}$$

where the non-cancer HQ is the ratio of exposure to hazardous substances, and RfD is the estimated maximum permissible risk that is imposed on humans through daily exposure. Experiencing adverse health effects is unlikely when HQ ≤ 1, whereas potential non-carcinogenic effects can occur when HQ > 1.

Moreover, the HQ calculated for each metal is summarized to assess the overall potential non-carcinogenic effects posed by more than one trace metal. If multiple pathways are available, a total exposure hazard index (HI) can be utilized to communicate non-cancer risks through different pathways:

(6) $$\mathrm{HI}=\overset{7}{\underset{i=1}{\sum }}{\mathrm{HQ}}_{i}$$

HI values > 1 shows that there is a chance that non-carcinogenic risk may occur; and when HI ≤ 1 the reverse applies.

Cancer risk (CR) and total cancer risk (TCR) can be evaluated from:

(7) $${\mathrm{CR}}_{i}=\overset{3}{\underset{j=1}{\sum }}{\mathrm{ADD}}_{\mathrm{ij}}\times {\mathrm{SF}}_{\mathrm{ij}}$$

(8) $$\mathrm{TCR}=\overset{2}{\underset{i=1}{\sum }}{\mathrm{CR}}_{i}$$

where cancer risk represents the probability of an individual lifetime health risk from carcinogens; SF is the slope factor of hazardous substances. The acceptable of tolerable risk for regulatory purposes is within the range of 10^−6^–10^−4^. In accordance with Agency for Research on Cancer, As and Cd are treated as having a potential carcinogen effect.

Human health risk from trace metals in multi-media are related to total metal concentrations, ingestion rate, and the bioavailability. In reality, trace metals in multi-media cannot be absorbed by humans to much of an extent. So in our study, total metal concentrations in multi-media will overestimate the residents’ potential risks.

### 2.5. Statistical Analysis 

Descriptive statistics were calculated by using the SPSS 20.0 software (IBM corporation, Armonk, NY, USA) and Excel 2013 (Microsoft corporation, Redmond, WA, USA). The data were displayed using the parameters of the minimum value, maximum value, mean value, the median, and standard deviation. Statistical analysis including Pearson correlation analysis, two significant levels at 5% and 1% were used in the statistics.

## 3. Results and Discussion

### 3.1. Trace Metals in Various Media and Local Crops

The trace metal concentrations in different media are presented in Table 1. The concentrations of seven metals in soil were much higher than the background values in Guangdong province. According to the Chinese Soil Environmental Quality Standard (GB15618-2008), mean concentrations of As (54.65 mg·kg^−1^), Cd (2.38 mg·kg^−1^), Pb (172.76 mg·kg^−1^), and Zn (332.53 mg·kg^−1^) were 2.73 times, 3.96 times, 3.46 times, and 1.11 times higher than the Grade II values, respectively. The mean concentrations of Cu (40.98 mg·kg^−1^), Ni (39.20 mg·kg^−1^), Cr (87.54 mg·kg^−1^) were blow the Grade II criteria (threshold value of soil contamination). Notably, even the minimum concentration of Cd (0.68 mg·kg^−1^) in the soils was higher than the Grade II values, indicating the severely contamination degree of Cd in the study area.

Six different species vegetable were collected in the field. When compared with the threshold values issued by Chinese Ministry of Health (GB2762-2012), except for the Pb contents in oil wheat (2.05 mg·kg^−1^), trace metal contents of vegetable (including leafy vegetable) were all under the food safety limits, which suggests that most vegetables have no potential risk in view of product quality. The DIs of trace metals for adults and children through food consumption are presented in Table 2. The DIs of trace metals in crops were in the following order: Zn > Cu > Ni > Cd > Pb > Cr > As. Intake of trace metals was much lower than the PTDI value. But, because staple food (rice, wheat) was not considered in this study, the results of DI would be limited.

The trace metal concentrations of surface water in the study area were lower than the standards for drinking water quality (GB5749-2006). However, the concentrations of As (50.17 mg·kg^−1^), Cd (14.83 mg·kg^−1^), Pb (247.77 mg·kg^−1^), and Zn (712.62 mg·kg^−1^) in sediments were much higher than the Chinese Soil Environmental Quality Standard (GB15618-2008). The high concentrations of trace meals in sediments may be because of the deposits of suspended particulates from the surface water, indicating that the water was polluted by the historical mining activities.

Trace metal concentrations in air deposition were higher than the average concentration of China level [37] and other regions [37,38]. Particularly, Pb and Zn concentrations in air deposition were much higher than those of other trace metals, suggesting that the accumulation of these two metals in soil may be mainly through fallout. There are three sampling locations set up in the north, middle and south of the study area, respectively. The results of ANOVA test showed that the interaction of sampling locations and months had no significant effect on the deposition rates by analyzing the trace metal content of each air sample during the four months. Monthly variations in deposition rates of trace metals were similar at different sampling locations, suggesting that trace metals deposition was mainly governed by local anthropogenic activities.

### 3.2. Trace Metal Concentrations in Human Hair

The bioaccumulation of trace metals in human hair is rather a complex process, factors that influence bioaccumulation include: nourishment, chemical forms of the metal and their binding site, age, sex, genetic inheritance, and environmental quality. In this study, the hair samples are classified into two groups of age (children (≤15 years) and adults (>15 years)).

The results of trace metals in the hair of inhabitants in different age ranges are represented in Table 3. The children group had much higher average concentrations of As (1.13 mg·kg^−1^), Cd (0.20 mg·kg^−1^), and Pb (10.88 mg·kg^−1^), about 1.59, 1.46, and 2.13 times higher than the adults group, respectively. The results indicate that children are sensitive to trace metal exposure, contaminated soil and dust may be ingested either directly or indirectly as a result of hand-to-mouth activity of children. The mean concentration of Zn (176.42 mg·kg^−1^) in group of adults was significantly higher than children. Food consumption was found to be an important source of endogenous deposition of trace metals to human hair. Although food requirements of adults are more than children, the need for trace elements of adults generally are less than children. Indicating that the change of growth rate may not parallel the rate of deposition of trace elements into hair [39].

In comparison with other regions in China and other countries [40,41,42], the levels of Pb and As in the hair of inhabitants were relatively high (Table 4). The mean concentration of Cu in hair samples was higher than the average level of other regions in China, but much lower than the mean level of Canada and USA inhabitants. When compared with the standard for hair’s trace metals of various Chinese populations, which were reported by the Trace Element Research Council of China (TERCC) [43], the average concentrations of trace metals in adults were between in the reference ranges. But, in children, the levels of As (1.13 mg·kg^−1^) and Pb (10.88 mg·kg^−1^) were higher than the upper limit value that was set by TERCC, indicating that children’s health has been affected by the trace metal contamination in the study area. Blood lead poisoning of local children were also reported in 2014 (local webpage). Therefore, it is necessary to control the Pb and As discharge of local mines and enterprises, and improve the risk perception of residents (especially children) on the prevention of trace metal contamination.

### 3.3. Health Risk Assessment

#### 3.3.1. Daily Exposure Levels

**Figure 2:**
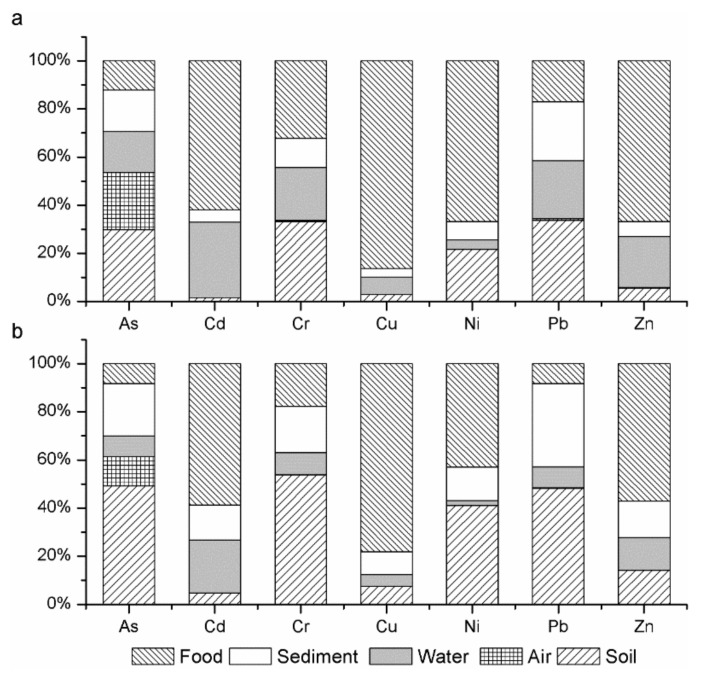
The contribution of Multi-media to the average daily exposure dose. (**a**) Each media’s contribution to the average daily dose of the local adults; (**b**) Each media’s contribution to the average daily dose of the local children.

Figure 2 showed the contribution of each exposure route to estimated ADD values of adults and children in the study area. For adults, food ingestion accounted for the majority of total ADD of Cd (61.84%), Cu (86.30%), Ni (66.83%), and Zn (66.84%), respectively. The intakes of the As and Pb in soil played the most important role of the total ADD, which accounted for 29.92% and 33.71%, respectively. For Cr, the ingestion pathway through food (32.23%) contributed nearly as much as the three pathway through soil (33.26%). For children, food ingestion contributed a major portion of the total ADD of Cd, Cu, and Zn, which accounted for 58.72%, 78.20%, and 57.09%, respectively. Soil plays the most important factor for As (49.27%), Cr (53.84%), and Pb (48.23%) intake. Soil provided 41.08% of the ADD of the Ni values nearly as much as food (42.97%). By contrast, air contributed less than 5% of ADD for adults and children, except for As. Thus, soil ingestion and food consumption are considered as the important pathways for trace metal exposure.

Furthermore, the correlation between the trace metal concentrations in hair and the average daily dose of trace metals via different exposure pathways was analyzed (Table 5). Hair trace metal accumulation were highly correlated with ingestion and inhalation pathways for both adults and children, indicating that human exposure to trace metals occurs predominately through ingestion and inhalation.

#### 3.3.2. Non-Carcinogenic Risks of Different Media

Table 6 summarized the non-carcinogenic risk assessment results for adults. The HI of trace metals decreased in the order of As > Cd > Pb > Cr > Cu > Zn > Ni. With regard to the pathways, the highest non-carcinogenic risk was from food ingestion, followed by the soil ingestion, water ingestion, and dermal contact with air. The adults’ HI of each metal was lower than 1, indicating that the potential health risk of individual metals was low. The majority of adults’ non-cancer risks was contributed by As (52.27%). Therefore, for the non-carcinogenic risks of adults, As should be received more attentions.

The results of non-carcinogenic risks of trace metals for local children through different exposure routes are shown in Table 7. Among the metals, As and Pb presented highest potential health risks, with the HI value being greater than 1. The children’s HQ of trace metals by soil ingestion (3.22), sediment ingestion (1.69) and crop ingestion (1.19) were higher than 1. Children had higher HQ via non-dietary ingestion of soil than adults because of more outdoor-playing times and hand-to-mouth activities (mouthing and chewing) [44,45]. According to other surveys, children accompany their parents to the farmland and are often exposed to the contaminated environment without any protective measures, which causes them easily be exposed to soil trace metals [46]. Therefore, Risk control should be taken about ingestion pathway to reduce the trace metal exposure level of children.

**Figure 3:**
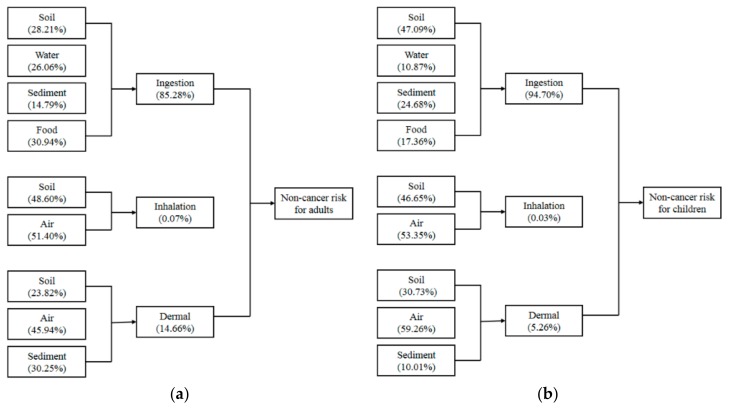
Multi-pathway analysis of hazard quotient (HQ). (**a**) Each pathway’s contribution to the total HQ of the local adults; (**b**) Each pathway’s contribution to the total HQ of the local children.

Figure 3 showed the contribution of each pathway to the health risk of local adults and children based on the average HQ values. Ingestion exposure of these trace metals largely contributed to the non-cancer effect, accounting for 85.28% and 94.70% to the total non-cancer risk of adults and children, respectively. Dermal contact was the second primary exposure route of all metals to the adults and children, which accounted for 14.66% and 5.26%, respectively. The effect of inhalation on human health can be ignored due to the low portion of the total non-cancer risks.

#### 3.3.3. Carcinogenic Risks of Different Media

Of the seven investigated elements, only As and Cd were considered highly carcinogenic. The proportion of different exposure routes, as indicated from the potential cancer risk assessments for adults and children, were asymmetrical (Table 8). The risk levels through inhalation from soil and air 10^−11^ to 10^−10^, much lower than the safe level, indicating that the cancer risk of inhalation can be ignored. Crops ingestion was the dominating exposure route to adults and children, which accounted for 47.30% and 40.16% of the total cancer risk, respectively. The cancer risk of Cd via water and crops ingestion for adults were 1.46 × 10^−4^ and 2.88 × 10^−4^, and the risk of Cd via crops ingestion for children was 1.89 × 10^−4^, were higher than the maximum safe value 10^−4^. The total cancer risk values of Cd for adults (4.66 × 10^−4^) and children (3.22 × 10^−4^) were much higher than those of As. Therefore, Cd appears to be the main pollutant source to produce cancer between the two elements. The cancer risk of As through ingestion and dermal contact pathways were all within the acceptable range of 10^−6^ to 10^−4^, existing some potential risk. Therefore, the most important step is to control the Cd pollution sources to prevent the further pollution, this would provide a powerful balance between environmental protection, crops safety improvement, reducing health risks, and massive savings of cost.

## 4. Conclusions

We investigated the distribution of trace metals in different environmental media and residents’ hair in a typical rural area affected by long term mining and industrial activities, and determined the potential health risk encountered through different exposure pathways. The main findings are:

(1) After long term mining and industrial activities, the area had been seriously polluted by trace metals, especially, As, Cd, and Pb, mainly through air deposition (for Pb) or irrigation (for As and Cd).
(2) Human hair is a suitable indicator for study the long-term metal exposure. Hair analysis results indicated that local resident, especially children, had been seriously affected by the trace metal contamination in the study area, and As and Pb concentrations in residents’ hair were relatively high.
(3) It also lead to high potential health risk. As and Pb presented highest potential non-carcinogenic health risks to children, with a HI value greater than 1, and the cancer risk of Cd exposure were higher than the maximum safe value 10^−4^.
(4) Risk control should be taken to reduce the ingestion exposure level of children, which was the dominated contribution to the non-cancer effect. Soil remediation should be conducted to reduce food and soil ingestion exposure.

By combining the accumulation of trace metals in residents’ hair with daily metal exposure value, we systematically analyzed the human health risk of trace metal through different pathways in multi-medias. The findings in our study suggest that local government should give priority to the soil remediation, and increase the risk perception of local children to reduce the trace metals exposure by hands to mouth activities. Further studies are needed in order to more accurately assess the trace metal intakes from soil and crops by local children.

## Figures and Tables

**Table 1:** Trace metal concentrations in different media in the study area.

Media	As	Cd	Cr	Cu	Ni	Pb	Zn
	Mean ± Std. Deviation (Minimum, Maximum)						
Soils (mg·kg^−1^)	54.65 ± 29.12	2.38 ± 2.22	87.54 ± 10.20	40.98 ± 12.67	39.20 ± 6.02	172.76 ± 106.76	332.53 ± 114.01
	(11.43, 175.73)	(0.68, 16.06)	(57.41, 114.75)	(20.05, 89.36)	(21.33, 62.03)	(89.84, 1258.29)	(118.11, 999.83)
Crops (mg·kg^−1^)	0.11 ± 0.11	0.30 ± 0.28	0.29 ± 0.29	4.35 ± 2.26	0.30 ± 0.45	0.42 ± 0.71	13.65 ± 6.90
	(0.01, 0.34)	(0.06, 1.27)	(0.01, 0.94)	(0.91,10.32)	(0.00, 2.79)	(0.01,3.82)	(1.37, 29.41)
Surface sediments (mg·kg^−1^)	50.17 ± 25.96	14.83 ± 9.81	62.93 ± 8.82	102.23 ± 49.32	26.8 ± 6.92	247.77 ± 115.18	712.62 ± 244.19
	(23.64, 87.18)	(2.17, 30.65)	(55.37, 75.85)	(39.22, 171.62)	(18.12, 35.78)	(101.32, 395.58)	(345.18, 986.58)
Surface water (μg·L^−1^)	2.09 ± 1.22	2.25 ± 4.31	2.94 ± 1.20	5.37 ± 4.37	0.36 ± 0.21	6.25 ± 14.60	63.75 ± 87.27
	(0.79, 5.34)	(0.16, 15.62)	(0.45, 5.25)	(1.75, 19.26)	(0.17, 0.91)	(0.52, 68.76)	(9.79, 329.40)
Air (μg·(m^2^·d)^−1^)	10.52 ± 4.74	0.40 ± 0.28	7.38 ± 6.31	11.27 ± 13.48	1.45 ± 1.11	30.85 ± 27.59	111.70 ± 118.11
	(5.02, 16.87)	(0.05, 0.72)	(1.58, 18.39)	(1.20, 38.12)	(0.25, 3.37)	(5.75, 82.92)	(14.18, 331.79)

**Table 2:** Dietary intake of trace metals (μg·day^−1^) for adults and children.

Daily Intake of Metals	As	Cd	Cr	Cu	Ni	Pb	Zn
Adults	3.23	8.80	8.50	127.56	8.80	12.32	400.29
Children	2.17	5.92	5.72	85.78	5.92	8.28	269.18
PTDI	580	70	500	6500	1200	200	33000

**Table 3:** Trace metals concentration of different age groups in human hair (mg·kg^−1^).

Age Group	Statistical Analysis	As	Cd	Cr	Cu	Ni	Pb	Zn
Children	Mean	1.13	0.19	0.17	11.43	1.21	10.88	120.70
	SD	0.64	0.07	0.06	2.35	0.36	3.28	29.62
Adults	Mean	0.71	0.13	0.21	11.37	1.21	5.118	162.997
	SD	0.36	0.06	0.13	2.65	0.526	3.020	51.803
Total	Mean	0.77	0.14	0.20	11.38	1.21	5.86	169.46
	SD	0.43	0.06	0.13	2.60	0.51	3.56	57.42

SD: standard deviation.

**Table 4:** Comparison of mean hair trace metal levels (mg·kg^−1^, dry weight) from the present study with those reported for other regions of the world.

Country/Region		As	Cd	Cr	Cu	Ni	Pb	Zn
Canada [40]		0.016	0.503	0.35	63.1	0.26	5.38	248
USA [40]		0.019	0.97	0.234	108	1.01	5.35	124
Japan [40]		0.053	0.28	0.23	10.7	2.7	3.62	114
Guangdong in China (Adults) [41]		0.77	0.052	0.34	NR	0.44	5.56	NR
Ningbo in China [42]		0.282	0.209	1.16	10.7	0.812	2.98	NR
Normal reference value by TERCC [43]	Adults	1.0	0.6	0.30–1.20	8.0–20.0	NR	10	120–210
	Children	1.0	0.5	0.18–0.72	8.0–16.0	NR	10	90–170

NR: not recommended. TERCC: Trace Element Research Council of China.

**Table 5:** Correlation coefficients between the concentrations of trace metals in hair and average daily dose of trace metals via different pathways.

Age Group	Ingest-Soil	Inhale-Soil	Dermal-Soil	Inhale-Air	Dermal-Air	Ingest-Water	Ingest-Sediment	Dermal-Sediment	Ingest-Crop
Adults	0.855 **	0.884 **	−0.015	0.959 **	−0.011	0.998 **	0.958 **	0.251	0.970 **
Children	0.903 **	0.905 **	−0.024	0.969 **	−0.021	0.999 **	0.972 **	0.245	0.970 **

Levels of significance: **: *p* < 0.01.

**Table 6:** Non-carcinogenic risk of trace metals for adults.

Type	Pathway	As	Cd	Cr	Cu	Ni	Pb	Zn	Total
Soil	Ingestion	2.82 × 10^−1^	3.68 × 10^−3^	4.51 × 10^−2^	1.58 × 10^−3^	3.03 × 10^−3^	7.63 × 10^−2^	1.71 × 10^−3^	4.13 × 10^−1^
	Inhalation	3.05 × 10^−5^	3.97 × 10^−7^	5.05 × 10^−4^	1.71 × 10^−7^	3.28 × 10^−7^	8.25 × 10^−6^	1.85 × 10^−7^	5.44 × 10^−4^
	Dermal contact	2.37 × 10^−2^	4.12 × 10^−3^	2.53 × 10^−2^	5.92 × 10^−5^	1.26 × 10^−4^	5.76 × 10^−3^	9.60 × 10^−4^	6.00 × 10^−2^
Air	Inhalation	1.01 × 10^−4^	6.37 × 10^−7^	4.58 × 10^−4^	4.96 × 10^−7^	1.29 × 10^−7^	1.54 × 10^−5^	5.72 × 10^−7^	5.76 × 10^−4^
	Dermal contact	7.45 × 10^−2^	6.26 × 10^−3^	2.17 × 10^−2^	1.62 × 10^−4^	4.68 × 10^−5^	1.02 × 10^−2^	2.81 × 10^−3^	1.16 × 10^−1^
Water	Ingestion	2.15 × 10^−1^	6.96 × 10^−2^	3.03 × 10^−2^	4.15 × 10^−3^	5.56 × 10^−4^	5.52 × 10^−2^	6.57 × 10^−3^	3.82 × 10^−1^
Sediment	Ingestion	1.29 × 10^−1^	1.15 × 10^−2^	1.62 × 10^−2^	1.98 × 10^−3^	1.04 × 10^−3^	5.47 × 10^−2^	1.84 × 10^−3^	2.17 × 10^−1^
	Dermal contact	2.17 × 10^−2^	2.57 × 10^−2^	1.82 × 10^−2^	1.48 × 10^−4^	8.60 × 10^−5^	8.25 × 10^−3^	2.06 × 10^−3^	7.61 × 10^−2^
Food	Ingestion	1.53 × 10^−1^	1.38 × 10^−1^	4.42 × 10^−2^	4.93 × 10^−2^	9.46 × 10^−3^	3.91 × 10^−2^	2.06 × 10^−2^	4.53 × 10^−1^
HI		8.99 × 10^−1^	2.59 × 10^−1^	2.02 × 10^−1^	5.74 × 10^−2^	1.43 × 10^−2^	2.50 × 10^−1^	3.66 × 10^−2^	1.72

HI: hazard index.

**Table 7:** The non-carcinogenic risk of trace metals for children.

Type	Pathway	As	Cd	Cr	Cu	Ni	Pb	Zn	Total
Soil	Ingestion	2.20	2.87 × 10^−2^	3.52 × 10^−1^	1.24 × 10^−2^	2.36 × 10^−2^	5.95 × 10^−1^	1.34 × 10^−2^	3.22
	Inhalation	6.16 × 10^−5^	8.04 × 10^−7^	1.02 × 10^−3^	3.47 × 10^−7^	6.63 × 10^−7^	1.67 × 10^−5^	3.75 × 10^−7^	1.10 × 10^−3^
	Dermal contact	4.61 × 10^−2^	8.03 × 10^−3^	4.93 × 10^−2^	1.15 × 10^−4^	2.45 × 10^−4^	1.12 × 10^−2^	1.87 × 10^−3^	1.17 × 10^−1^
Air	Inhalation	2.21 × 10^−4^	1.39 × 10^−6^	1.00 × 10^−3^	1.08 × 10^−6^	2.82 × 10^−7^	3.36 × 10^−5^	1.25 × 10^−6^	1.26 × 10^−3^
	Dermal contact	1.45 × 10^−1^	1.22 × 10^−2^	4.24 × 10^−2^	3.17 × 10^−4^	9.13 × 10^−5^	1.98 × 10^−2^	5.48 × 10^−3^	2.25 × 10^−1^
Water	Ingestion	4.20 × 10^−1^	1.36 × 10^−1^	5.91 × 10^−2^	8.09 × 10^−3^	1.08 × 10^−3^	1.08 × 10^−1^	1.28 × 10^−2^	7.44× 10^−1^
Sediment	Ingestion	1.01	8.95 × 10^−2^	1.27 × 10^−1^	1.54 × 10^−2^	8.08 × 10^−3^	4.27 × 10^−1^	1.43 × 10^−2^	1.69
	Dermal contact	1.09 × 10^−2^	1.28 × 10^−2^	9.08 × 10^−3^	7.38 × 10^−5^	4.30 × 10^−5^	4.13 × 10^−3^	1.03 × 10^−3^	3.81 × 10^−2^
Crop	Ingestion	4.00 × 10^−1^	3.62 × 10^−1^	1.16 × 10^−1^	1.29 × 10^−1^	2.48 × 10^−2^	1.03 × 10^−1^	5.41 × 10^−2^	1.19
HI		4.23	6.48 × 10^−1^	7.56 × 10^−1^	1.66 × 10^−1^	5.80 × 10^−2^	1.27	1.03 × 10^−1^	7.23

**Table 8:** Carcinogenic risk of trace metals for adults and children.

Age Group	Type	Pathway	As	Cd	Total
Adults	Soil	Ingest	4.35 × 10^−5^	7.69 × 10^−6^	5.12 × 10^−5^
		Inhale	1.35 × 10^−11^	5.18 × 10^−11^	6.52 × 10^−11^
		Dermal	1.46 × 10^−5^	8.61 × 10^−8^	1.47 × 10^−5^
	Air	Inhale	4.47 × 10^−11^	8.30 × 10^−11^	1.28 × 10^−10^
		Dermal	4.59 × 10^−5^	1.31 × 10^−7^	4.61 × 10^−5^
	Water	Ingest	3.32 × 10^−5^	1.46 × 10^−4^	1.79 × 10^−4^
	Sediment	Ingest	2.00 × 10^−5^	2.40 × 10^−5^	4.39 × 10^−5^
		Dermal	1.34 × 10^−5^	5.37 × 10^−7^	1.39 × 10^−5^
	Crop	Ingest	2.35 × 10^−5^	2.88 × 10^−4^	3.12 × 10^−4^
	TCR		1.94 × 10^−4^	4.66 × 10^−4^	6.60 × 10^−4^
Children	Soil	Ingest	8.48 × 10^−5^	1.50 × 10^−5^	9.97 × 10^−5^
		Inhale	6.82 × 10^−12^	2.62 × 10^−11^	3.30 × 10^−11^
		Dermal	7.12 × 10^−6^	4.20 × 10^−8^	7.16 × 10^−6^
	Air	Inhale	2.44 × 10^−11^	4.54 × 10^−11^	6.99 × 10^−11^
		Dermal	2.24 × 10^−5^	6.38 × 10^−8^	2.25 × 10^−5^
	Water	Ingest	1.62 × 10^−5^	7.09 × 10^−5^	8.71 × 10^−5^
	Sediment	Ingest	3.89 × 10^−5^	4.68 × 10^−5^	8.57 × 10^−5^
		Dermal	1.68 × 10^−6^	6.72 × 10^−8^	1.74 × 10^−6^
	Crop	Ingest	1.54 × 10^−5^	1.89 × 10^−4^	2.04 × 10^−4^
	TCR		1.86 × 10^−4^	3.22 × 10^−4^	5.08 × 10^−4^

TCR: total cancer risk.

## Supplementary Materials

Supplementary File 1
