# Supplementary material for: Health Risk Assessment of Trace Metals in Various Environmental Media, Crops and Human Hair from a Mining Affected Area
Source: Int J Environ Res Public Health. 2017 Dec 18;14(12):1595. doi: 10.3390/ijerph14121595 (PMC5751012; doi:10.3390/ijerph14121595)
Supplement: Supplementary File 1 [file ijerph-14-01595-s001.pdf]

Supporting Information

Table S1. Soil environmental quality standard (GB15618-2008) and background value (mg·kg^{-1}).

(Please add the column heading.)

Column Heading	pH	As	Cd	Cr	Cu	Ni	Pb	Zn
GB15618-2008	≤5.5	35	0.25	120	150	60	50	150
	5.5–6.5	30	0.3	150	150	70	50	200
	6.5–7.5	25	0.45	200	200	80	50	250
	>7.5	20	0.6	200	200	90	50	300
Background	5.2	8.9	0.056	50.5	17	14.4	36	47.3

Table S2. Maximum levels of Contaminants in Foods (GB 2762-2012) (mg·kg^{-1}, fresh weight).

As	Cd	Cr	Cu	Ni	Pb	Zn
0.5	0.1 corn	1.0 corn	NR	NR	0.2 corn	NR
	0.2 leaf vegetable	0.5 fresh vegetable			0.3 leaf vegetable	
	0.1 root vegetable				0.1 fresh vegetable	
	0.05 fresh vegetable					

NR: not recommended

Table S3. Maximum levels of contaminants in irrigation water (GB5749-2006) (mg·L^{-1}).

As	Cd	Cr	Cu	Ni	Pb	Zn
0.05	0.01	0.1	1.0	0.1	0.2	2.0

Table S4. Parameter values in average daily does calculation models of heavy metals. (Please confirm the unite "d·a^{-1}".)

Item	Parameter	Value
ADD_{ing}	InR/ mg·d^{-1} (soil)	200 (children), 100 (adults)
	InR/ mg·d^{-1} (crops)	232×10^3 (children), 345×10^3 (adults)

	InR/ $L\cdot d^{-1}$ (water)	1.0 (children), 2.0 (adults)
	InhR/ $m^3\cdot d^{-1}$ (soil)	7.63 (children), 14.7 (adults)
ADD_{inh}	InhR/ $m^3\cdot d^{-1}$ (air)	8.7 (children), 15.5 (adults)
	PEF/ $m^3\cdot kg^{-1}$	1.36×10^9
ADD_{derm}	SA/cm^2	2800 (children), 16000 (adults)
	AF/$mg\cdot(cm^2\cdot d)^{-1}$	0.2 (children), 0.07 (adults)
	ABS	0.03 (As), 0.001 (other metals)
Exposure pathway parameters	ED/a	6 (children), 24 (adults)
	EF/$d\cdot a^{-1}$	350
	BW/kg	15.9 (children), 62.0 (adults)
	AT/d	ED×365(non-carcinogens)<br>70×365 (carcinogens)
	CF/$kg\cdot mg^{-1}$	1×10^{-6}

Note: InhR is the inhalation rate, PEF is the particle emission factor, SA is the surface area of skin exposed to pollutants, AF is the skin adherence factor, ABS is the dermal absorption factor, EF is the exposure frequency, ED is the exposure duration, BW is the body weight, and AT is the average time for non-carcinogens or carcinogens, CF is the units conversion factor.

Table S5. References does for non-carcinogen metals and slope factors for carcinogen metals.

Item	As	Cd	Cr	Cu	Ni	Pb	Zn
RfD_{ing}/ $mg\cdot(kg\cdot d)^{-1}$	0.0003	0.001	0.003	0.04	0.02	0.0035	0.3
RfD_{inh}/ $mg\cdot(kg\cdot d)^{-1}$	0.0003	0.001	0.000029	0.04	0.02	0.0035	0.3
RfD_{derm}/ $mg\cdot(kg\cdot d)^{-1}$	0.0012	0.00001	0.00006	0.012	0.0054	0.00052	0.006
SF_{ing}/ $(kg\cdot d)\cdot mg^{-1}$	1.5	6.1					
SF_{inh}/ $(kg\cdot d)\cdot mg^{-1}$	0.0043	0.38					
SF_{derm}/ $(kg\cdot d)\cdot mg^{-1}$	1.5	6.1					

Local webpage

Website: https://news.qq.com/a/20141108/003391.htm

Title: Blood lead poisoning of 16 children in Wengyuan county of Guangdong province due to the companies' pollution emission.

广东翁源16名儿童血铅超标 村民称因企业排污

各地新闻 | 南方报业网-南方农村报 王伟正 2014-11-08 03:40 | 我要分享

0

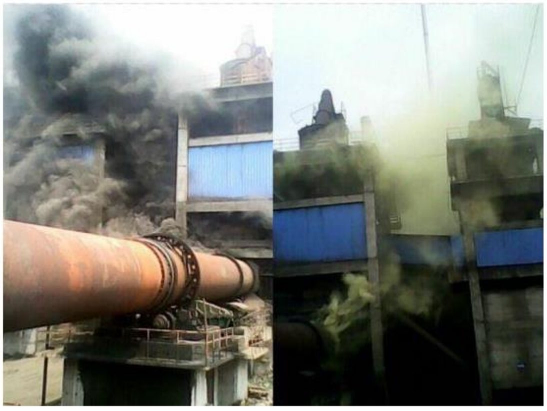

铁龙工厂废气排放

发送图片到手机

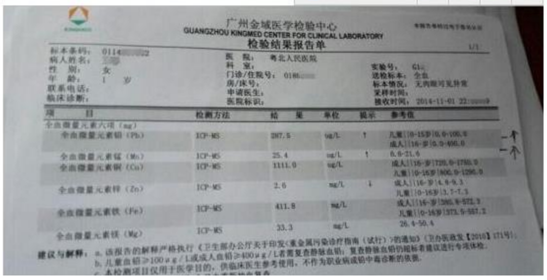

广州金域医学检验中心
GUANGZHOU KINGMED CENTER FOR CLINICAL LABORATORY
检验结果报告单

1/1

标本条码：0114[illegible]2
病人姓名：[illegible]
性 别：女
年 龄：1 岁
联系电话：
临床诊断：
医 院：粤北人民医院
科 室：
门诊/住院号：0186[illegible]
房/床号：
申请医生：
医院标识：
实验号：G1[illegible]
送检标本：全血
标本情况：无肉眼可见异常
采样时间：
接收时间：2014-11-01 22:[illegible]

项 目	检测方法	结 果	单位	提示	参考值
全血微量元素六项（mg）					
全血微量元素铅（Pb）	ICP-MS	287.5	ug/L	↑	儿童||0-15岁|0.0-100.0 成人||16-岁|0.0-400.0
全血微量元素锰（Mn）	ICP-MS	25.4	ug/L	↑	6.6-21.6
全血微量元素铜（Cu）	ICP-MS	1111.0	ug/L		成人||16-岁|720.0-1750.0 儿童||0-16岁|806.0-1290.0
全血微量元素锌（Zn）	ICP-MS	2.6	mg/L	↓	成人||16-岁|4.8-9.3 儿童||0-16岁|3.7-7.3
全血微量元素铁（Fe）	ICP-MS	411.8	mg/L		成人||16-岁|395.8-572.3 儿童||0-16岁|373.5-557.2
全血微量元素镁（Mg）	ICP-MS	33.3	mg/L		26.4-50.4

建议与解释：a. 该报告的解释严格执行《卫生部办公厅关于印发〈重金属污染诊疗指南（试行）〉的通知》（卫办医政发【2010】171号）；
b. 儿童血铅≥100μg/L或成人血铅≥400μg/L者需复查静脉血铅，复查静脉血铅仍超标者建议进行专项体检。不作为职业病或铅中毒诊断的依据。
c. 本检测项目仅用于医学目的，供临床医生参考使用[illegible]
